# Prospective approaches to enhancing CAR T cell therapy for glioblastoma

**DOI:** 10.3389/fimmu.2022.1008751

**Published:** 2022-10-06

**Authors:** Sun Il Choi, Jinlong Yin

**Affiliations:** ^1^ Henan Key Laboratory of Brain Targeted Bio-Nanomedicine, School of Life Sciences & School of Pharmacy, Henan University, Kaifeng, China; ^2^ Henan-Macquarie University Joint Centre for Biomedical Innovation, School of Life Sciences, Henan University, Kaifeng, China

**Keywords:** glioblastoma, cancer therapy, cell therapy, immunotherapy, CAR T

## Abstract

Glioblastoma (GBM) is the most common malignant brain tumor. The poor clinical outcome and overall ineffectiveness of current standard treatments, including surgery, chemotherapy, and radiation, highlight the urgent need for alternative tumor-specific therapies for GBM. Chimeric antigen receptor (CAR) T cell therapy is a revolutionary therapeutic strategy for hematological malignancies, but the optimal potency of CAR T cell therapy for solid tumors, especially GBM, has not been achieved. Although CAR T cell therapeutic strategies for GBM have been assessed in clinical trials, the current antitumor activity of CAR T cells remains insufficient. In this review, we present our perspective on genetically modifying CAR constructs, overcoming T cell dysfunctions, and developing additional treatments that can improve CAR T cell effectiveness, such as functionality, persistence, and infiltration into tumor sites. Effectively improved CAR T cells may offer patients with GBM new treatment opportunities, and this review is intended to provide a comprehensive overview for researchers to develop potent CAR T cells using genetic engineering or combinatorial preparations.

## Introduction

**Figure 1:**
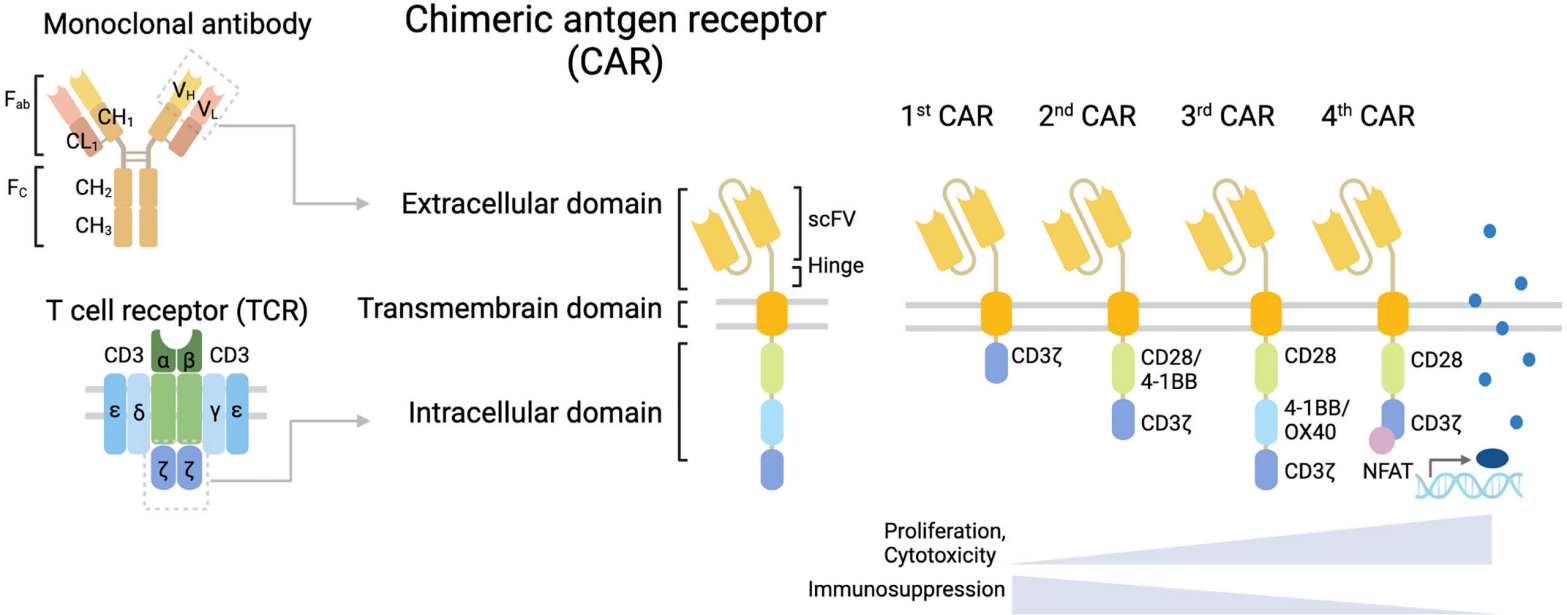
Structure of CAR T cell. CAR T cells have a structural design with three major domains: an extracellular domain including a hinge (space), a transmembrane domain, and an intracellular signaling domain. First-generation CARs include a single-chain variable fragment (scFv) targeting tumor-associated antigen (TAA) linked to a space in an extracellular domain, a transmembrane domain, and the CD3ζ chain of the T cell receptor (TCR) as an intracellular signaling domain. Second-generation CARs are enhanced CAR T cell activity by adding a costimulatory domain, such as CD28 or 4-1BB to support the expansion and persistence of engineered T cells. Third-generation CARs contain several costimulatory domains.

Chimeric antigen receptor (CAR) T cell therapy has emerged due to the development of genetically engineered T cell receptors (TCRs) to achieve specific target cancer-associated antigens (**Figure 1**). Adoptive transfer of autologous CAR T cells prolongs patient survival times and promotes remission, even in some patients who do not respond to standard treatment (1). Since the first CD19-CAR T cell therapy for patients with acute lymphoblastic leukemia (ALL) was approved by the FDA in 2017, investigators have concentrated on extending the therapeutic effects of CAR T cells not only for B cell malignancies but also for several other cancers, including solid tumors.

**Figure 2:**
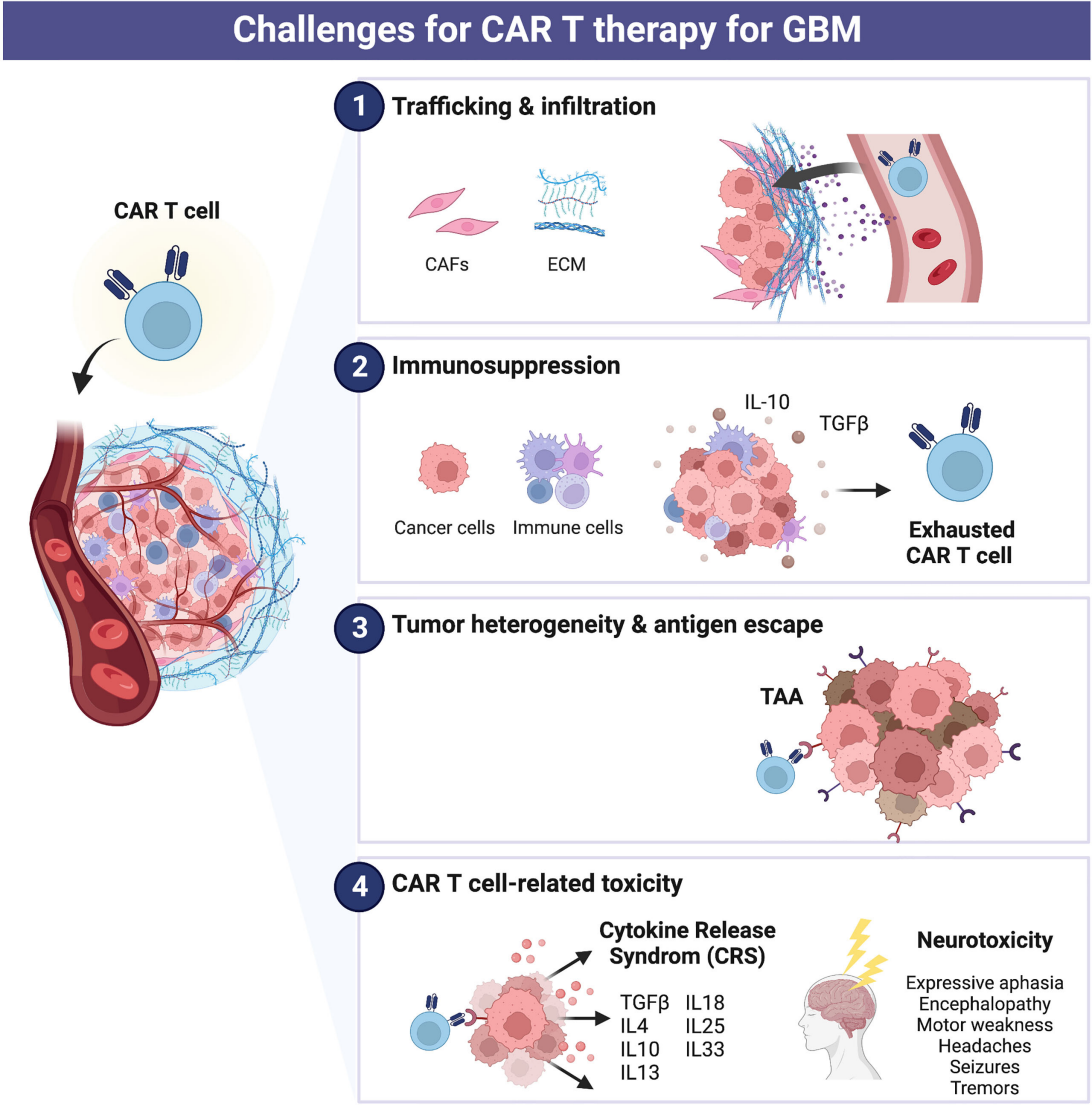
Challenges for CAR T therapy for glioblastoma (GBM). Current major limitations of CAR cell therapy include (1) trafficking and infiltration, (2) immunosuppression, (3) tumor heterogeneity and antigen escape, and (4) CAR T cell-related toxicity.

Glioblastoma (GBM), the most common and highly aggressive malignant brain tumor, has a poor prognosis with a median overall survival time of <16 months (2, 3). Currently, the approved GBM treatments typically include surgery followed by radiotherapy or chemotherapy, with a 5-year survival rate below 10%. Several immunotherapeutic approaches have been examined in clinical trials for patients with GBM. However, the impact of CAR T cells on early clinical outcomes was limited as the cells did not generate sufficient antitumor activity, and there is still no FDA-approved CAR T therapy for GBM despite safe and promising results (4–7). The development and research of CAR T cell therapy against GBM remains challenging to date due to complications, including antigen heterogeneity, loss of cells rendering CAR obsolete (T cell exhaustion), immunosuppressive molecules and cells in the tumor microenvironment (TME), and insufficient CAR T cells penetration and trafficking to tumor sites (**Figure 2**) (8–11).

**Figure 3:**
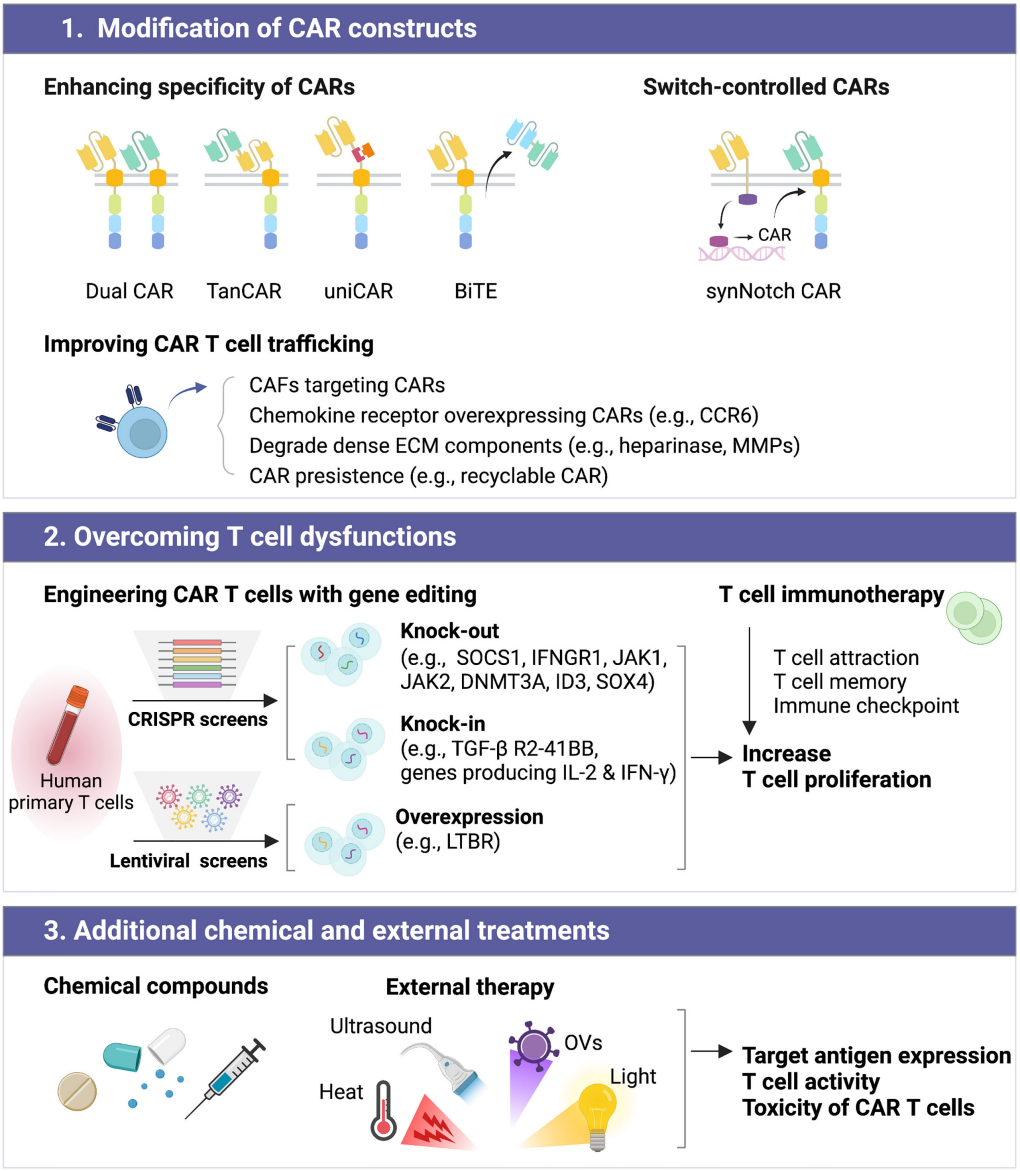
Improving the efficacy of CAR T cell therapy can be classified into distinct subgroups, including (1) modification of CAR constructs, (2) overcoming T cell dysfunctions, and (3) additional chemical and external treatments.

Here, we review current and emerging strategies in the field of CAR T cell therapy with a focus on the following three aspects (1): modification of the CAR construct, (2) overcoming T cell dysfunctions, and (3) the addition of chemical and external treatments (**Figure 3**). We discuss the individual strength of these technologies and their applications for GBM therapy. Although potential barriers to CAR T cell therapy still exist, effectively improved CAR T cell therapy could offer patients with GBM new treatment opportunities. This review provides a comprehensive summary of individual strengths for those aiming to develop more advanced CAR T cells using genetically modified constructs or combinatorial preparations.

## Modification of CAR constructs

CAR T cell structure comprises three major domains: an extracellular domain, a transmembrane domain, and an intracellular signaling domain (**Figure 1**). Each domain has a unique function and requires an optimal design for CAR efficacy. Additionally, CAR designs have been adjusted to improve clinical effectiveness, and new design approaches are emerging. Indeed, the CAR T cells currently under development have been designed by genetically modifying the CAR construct to compensate for the weakness of existing CAR T cells, such as poor activity against GBM or severe toxicity caused by CAR T cell overactivation.

### Enhancing the specificity of CARs for GBM tumors

One of the reasons for the poor activity of GBM CAR T cells is the low target specificity of CARs. Although IL-13Rα2, EGFRvIII, and HER2 are the most frequently targeted antigens in preclinical and clinical studies on CAR T therapy for GBM, these antigens have important limitations (4–7). Therefore, several approaches have been investigated to overcome the antigen heterogeneity and immune escape leading to loss of antigenicity and/or immunogenicity when a single antigen is targeted in GBM, and some of these approaches reflect the strategies used for hematological malignancies.

Researchers have designed CAR T cells to have two CARs, each targeted to a different type of tumor-related antigens (DualCARs) or assembled two or more scFvs linearly (tandem CARs; TanCARs), similar to the bispecific antibody platform with dual affinity retargeting (12). However, currently, CD19/CD22- (13) and CD19/CD20-targeting approaches such as these CARs have limited the clinical experience for leukemia therapy (7, 14). Another approach is to design CARs that split from CAR molecules to the targeting domain, which is capable of binding multiple undefined antigens (universal CAR; uniCAR), and as a typical example, the T1E peptide binding to EGFR family receptors is the most relevant example for solid tumors (15). Moreover, bispecific T cell engagers (BiTEs) platforms have recently been developed capable to target multiple antigens with flexibility for combinatorial and sequential approaches and redirecting T cells activity toward tumor cells. Furthermore, other strategies are to design T cells capable of recognizing tumor cells by inducing bystander T cell activation, including the transgenic expression of cytokines (e. g., IL-18 or IL-36γ) or ligands (e.g., CD40L) (16). Similarly, one study revealed that engineered T cells to secrete the dendritic cell (DC) growth factor Fms-like tyrosine kinase 3 ligand (Flt3L) induces host T cell activation (17). In addition, in mouse GBM models, anti-Fn14×CD3 BiTE given intratumorally showed antitumor effects whereas xenografts treated with Fn14 CAR T cells recurred (18). Furthermore, to address antigenic heterogeneity in GBM, investigators have developed gene modifying EGFRvIII CAR T cells to deliver bispecific EGFR antibodies (EGFRvIII CAR T BiTEs) (19).

### Switch-controlled CARs for reducing on-target/off-tumor toxicity

Most antigens targeted by CAR T cells are tumor-associated rather than tumor-specific, which means highly expressed on the tumor and have low abundance in normal tissues, capable of leading to providing on-target/off-tumor toxicities. Much research has been conducted on switch-controlled CARs, which can solve these problems by controlling activation to kill tumors while providing inhibitory mechanisms such as a CAR T cell suicide activation and methods to regulate the costimulatory pathway.

One of the best switch-controlled CAR T cells is using an antigen-specific synthetic notch (synNotch) system, conferring the expression of a second CAR upon activation (20). Numerous investigators have reported SynNotch gated CAR-T cells, such as SynNotch-GD2-B7H3, which recognize GD2 as the gate and B7H3 as the target (21), and SynNotch-HER2 (22), which controls the killing effects. The low-affinity synNotch receptor performs as a filter, limiting transcriptional induction until T cells encounter high antigen-expressing target cells. Once passed through this initial filter, the induced high-affinity CAR can perform potent T cell proliferation and killing activities. This synNotch CAR low-high circuit design allows CAR-T cells to express CAR molecules only in the presence of tumor cells with high antigen density, thereby killing tumor cells but not healthy cells with low antigen density.

In addition, two synNotch CAR researchers recently used a different approach in which stable levels of activity were maintained throughout the anticancer process, eliminating the problem of T cell depletion that has hampered traditional CAR T therapy (23, 24). They pursued two such multiantigen targeting strategies for GBM, EGFRvIII-SynNotch CAR and EphA2 or IL13Ra2 CARs (23), and approaches for mesothelioma and ovarian cancer involving alkaline phosphatase placental-like 2 (ALPPL2)-synNotch CAR and MCAM, MSLN or HER2 CAR (24). Traditional CAR T cells are designed to consistently express a kill switch; however, in these two SynNotch CAR T cells can save energy by going into standby mode when not in direct contact with the tumor.

### Improving CAR T cell trafficking

CAR T cells for hematological malignancies and their corresponding target malignancies share a hematopoietic origin and therefore tend to migrate to similar areas, such as the bone marrow or lymph nodes. In comparison, most solid tumors, including GBM, do not readily attract T cells and are known as cold tumors due to their poor immunogenic status. Therefore, CAR T cells for GBM should be engineered to increase CAR T cell trafficking, allowing them to penetrate the extremely dense stroma and be attracted to tumors.

Stromal cells, such as cancer-associated fibroblasts (CAFs), express high levels of fibroblast activation protein (FAP) (25) and SLAMF7 (26) and induce remodeling of the aberrant extracellular matrix (ECM), which limits T cell motility and trafficking. Some groups have reported that combination treatment with FAP-targeting CAR T cells and tumor-targeting CAR T cells or cancer vaccines showed enhanced antitumor immunity, but there are conflicting results regarding FAP-targeting CAR T cells, which had on-target, off-tumor toxicity against bone marrow stromal cells (8). Thus, targeting stromal cells expressing FAP requires further investigation of the efficacy and toxicity profile of FAP-targeted CAR T cells (10). Meanwhile, dual-targeting CAR T cells for both malignant plasma cells and CAFs showed a potential ability to reverse TME-induced CAR T cell suppression (26).

Moreover, chemokine receptor-overexpressing CAR T cells can potentially be attracted to tumors that highly express chemokine receptors, such as CCR6, leading to effective tumor clearance (27, 28). Additionally, in parallel, technologies to enable *in vivo* real-time imaging of CAR T cells are being generated and assessed in preclinical and early phase clinical trials (29, 30). Such a strategy would enable the noninvasive and rapid examination to improve CAR T cell trafficking to tumor cells and antitumor function.

Furthermore, another approach is to efficiently degrade dense ECM components to promote CAR T cell infiltration to the tumor and its antitumor activity. Researchers have attempted to engineer CAR T cells with enzymes that degrade dense ECM with developed stiffness and crosslinking, such as heparinase (which degrades heparan sulfate proteoglycan) and matrix metalloproteinases (MMPs; which proteolysis all ECM components in a Zn-dependent manner) (8, 31).

In addition, CAR persistence is also important for CAR T cell trafficking. For example, CAR ubiquitination is triggered during CAR encounters with tumor antigens. Thereby, ubiquitination-blocked recyclable CAR, mutating the intracellular ubiquitination site (CAR^KR^), could elevate endosomal CAR signaling and be recyclable back to the cell surface, leading to redirecting CAR trafficking (32).

## Overcoming T cell dysfunctions

Most patients, except for B lymphoblastic leukemia patients, do not show a sustained response to CAR T cells and develop resistance mainly causing T cell dysfunction (33, 34). Substantial efforts have been made to identify genes and pathways contributing to T cell dysfunction. In this section, we explain the modulation of CAR T cell function by structural engineering, leading to premature CAR T cell differentiation and the prevention of exhaustion.

### Engineering CAR T cells with gene editing

The utility of CRISPR-Cas9 genome engineering, in other words, loss-of-function screening, has developed it possible to easily knock-out any gene in the genome with extension and customization. Deleting or inhibiting some genes enhances CAR T cell activity (35, 36). Several investigators have identified a gene signature that defines CAR and TCR dysregulation and transcription factors as key regulators of CAR T cell exhaustion using CRISPR-Cas9 screens. In CD19 CARs in CD4^+^ or CD8^+^ T cells with deletion of suppressor of cytokine signaling 1 (SOCS1), a major nonredundant checkpoint that inhibits CD4^+^ T cell proliferation by blocking multiple downstream signaling molecules, both IL-2 and IFN-γ, results in a markedly improved antitumor effect (37). Additionally, loss of genes in the IFN-γR signaling pathway (IFNGR1, JAK1, or JAK2) reduces overall CAR T cell binding duration and avidity in GBM, with other solid tumors being more resistant to CAR T cell-mediated death (38). The deletion of the *de novo* DNA methyltransferase 3 alpha (DNMT3A) in first- or second-generation CAR T cells universally preserved the cell proliferation and antitumor response during prolonged tumor exposure (39). The exhaustion-resistant DNMT3A knock-out CAR T cells have been coupled with the upregulation of IL-10, and genome-wide DNA methylation profiling revealed an atlas of genes targeted for epigenetic silencing (39). Moreover, the deletion of ID3 or SOX4 may attenuate cytotoxicity, relative to a CD8^+^ T to-NK-like T cell transition (NK-like CAR T cells), suggesting a potential strategy to enhance the efficacy of CAR T cell therapy against solid tumors (33). Furthermore, knocking out transforming growth factor-β receptor II (TGF-βRII) in CAR *via* CRISPR promotes the long-term efficacy of CAR T cells against solid tumors (40). Recently, the results of the phase I trials have revealed that prostate-specific membrane antigen (PSMA) CAR T optimally engineered with TGF-βRII to block TGF-β signaling to demonstrate improved T cell expansion in blood, tumor trafficking, and enhanced antitumor immunity in metastatic castration-resistant prostate cancer (41). These atlases enhance CAR T cell efficacy and provide a molecular definition of CAR T cell exhaustion.

Advances in CRISPR technology have also allowed for the investigation of T cell function beyond loss-of-function screens, improving our understanding of the regulators of T cell activation as a result of gain-of-function gene perturbations and providing further insight into disease pathways. One research group utilized a widely adaptable technology for barcoding and tracking targeted integrations of large-scale nonviral DNA templates for pooled knock-in screens in primary human T cells and pooled knock-in sequencing (PoKI-seq), combining single-cell transcriptome analysis *in vitro* and *in vivo* (42). Through this platform, they engineered transforming growth factor-β (TGF-β) R2-41BB chimeric receptor to promote solid tumor clearance (42). Another group reported using genome-wide CRISPR activation (CRISPRa) and interference (CRISPRi) screens to construct gene networks regulating IL-2 and IFN-γ production in primary human T cells (43). Alterations in the cytokine response were confirmed through key hits by arrayed CRISPRa and multiplexed secretome characterization. Combining CRISPRa screening with single-cell RNA sequencing for deep molecular characterization of screened hits is possible, demonstrating how perturbations modulate T cell activation and promote cellular states characterized by notable cytokine expression profiles. The genes involved in reprogramming critical immune cell functions identified through these screens might be applied in the design of immunotherapies.

Although CRISPR screens offer complexity and diversity, lentiviral screens have readily been applied for the T cell phenotype and are well established, suggesting opportunities for clinical translation. For instance, one research group achieved a genome-wide gain-of-function screen in primary human CD8^+^ and CD4^+^ T cells by applying barcoded human open reading frames (ORFs) and identified the top-ranked genes and key cytokines (44). From these results, the lymphotoxin-β receptor (LTBR), which is the high-ranked ORF, causes profound transcriptional and epigenomic remodeling, activating the NF-κB pathway in overexpressed T cells, thereby increasing T cell effector functions in the chronic stimulatory environment and inducing depletion, i.e., the antigen-specific responses of CAR T cells were improved.

### Application of T cell immunotherapy

Several approaches to T cell immunity have been published recently. After chemotherapy with cisplatin, producing CCL20 and IL-1β at the tumor site induces to recruiting and activating innate lymphoid cells (ILC3s) in tumors. Then, activated ILC3s generate CXCL10, which attracts CD4^+^ and CD8^+^ T cells to tumors, enhancing antitumor immunity (45). Moreover, the approved pharmacologic inhibitors of cyclin-dependent kinases 4 and 6 (CDK4/6) promote the phenotypic and functional acquisition of immunologic T cell memory (46). This applies to the design of clinical trial protocols to prevent CAR T cell exhaustion during therapy. Indeed, one research group identified protein tyrosine phosphatase 1B (PTP1B) as an intracellular checkpoint that is upregulated in T cells in tumors; additionally, they revealed that deletion or inhibition of PTP1B improves the efficacy of adoptively transferred CAR T cells against solid tumors (47). Their hypothesis rationalized the relationship between cytokine-induced JAK/STAT signaling, which is well established to be fundamentally important in all aspects of immunity, particularly in T cell activation and homeostasis, and PTP1B, which can attenuate this signaling. Such successful immunotherapy cases, theories, and evidence can be actively implemented for the development of CAR T cell treatments.

Overall, transient suspension of CAR signaling molecules and receptor redesign have been proposed to improve antitumor activity. Alternatively, signaling levels might be reduced, allowing for more controlled and dynamic regulation of CARs, similar to the natural T cell receptor.

## Additional chemical and external treatments

### Chemical compounds for CAR T cell therapy

Chemical compounds can increase target antigen expression or T cell activities and have a synergetic effect on CAR T cells. The enhancer of zeste 2 polycomb repressive complex 2 (Ezh2) subunit inhibitor has upregulated GD2 expression and enhanced GD2 CAR T efficacy in Ewing sarcoma (48). Sunitinib, a multi-tyrosine kinase inhibitor, has upregulated carbonic anhydrase IX (CAIX) expression and improved the efficacy of anti-CAIX CAR T cells in renal cancer (49). Bryostatin1, a protein kinase C modulator, and its analogs were shown to upregulate CD22 to enhance CD22-CAR T cell efficacy (50, 51). The pharmacologically selective inhibition of PTP1B using MSI-1436 induced the T cell-mediated tumor suppression and enhanced PD-1 blockade response (47). Moreover, a combination of anti-PD-1-based immunotherapy with IFN-α resulted in encouraging anticancer activity in unresectable HCC patients (52). In infiltrating CD8^+^ T cells, IFN-α downregulates the ability to consume glucose and consequentially establishes a high glucose microenvironment promoting the transcription of CD27, which is the T cell costimulatory molecule.

By reversibly binding to CAR T cells, small compounds also play a safety switch role to address the toxicity of CAR T cell therapy by reversibly binding to CAR T cells. In lymphoma, the clinically approved drug dasatinib, which inhibits the phosphorylation of CD3, LCK, and ZAP70, results in restricted induction of NFAT and induces CD8^+^ and CD4^+^ CAR T cells to be a function-off state (53). Lenalidomide intercedes the proteasomal degradation of several target molecules through modulation of CRL4^CRBN^ E3 ubiquitin ligase and a C2H2 zinc finger degron motif (54) that functions as a switch molecule, has been shown to be a safe induction agent for genetic vehicles designed for therapeutic uses. Duong’s research group developed dual-switch CAR T cells in which one is a potent activation switch based on rimiducid-inducible MyD88 and CD40 (iMC)-signaling elements to improve CAR T cell efficacy, and the other is an orthogonally regulated, rapamycin-induced, caspase-9-based safety switch (iRC9) to neutralize potential toxicity by this reinforced CAR (55). Similarly, Yang’s research group constructed two types of CAR T cells with the stilbenoid natural product resveratrol (3,4’,5-trihy-droxystilbene) as a switch molecule, containing gene circuits that can control the activation (on) and inactivation (off) of CAR T cells (56). These tunable dual-switch systems provide a safety switch to reduce toxicity, thus supporting greater CAR T cell expansion and long-term persistence in a drug-dependent manner.

Small molecule compounds such as those mentioned above are likely sufficiently safe; preferably, such compounds have low immunogenicity and clear pharmacodynamic and pharmacokinetic properties. Small molecule compounds should also be selectively reached to target tumor tissues *via* drug-directed delivery techniques, further mitigating off-target toxicity. Meanwhile, several compounds with CAR T cells have already been demonstrated to exhibit synergistic effects in clinical trials. For example, B-cell lymphoma patients achieved complete remission following a combination of anti-CD19 CAR T cells and decitabine (57). A case study revealed that CD19-CAR T cell therapy with dasatinib induced complete remission in lymphoid blast phase chronic myeloid leukemia harboring the T315I mutation in the BCR-ABL fusion gene (58). Moreover, combination CAR T cell therapy following ibrutinib in patients with relapsed or refractory CLL demonstrated the probability of one-year progression-free survival and lower CRS-associated cytokines in serum (59). Recently, the high-performance drug-regulatable system termed signal neutralization by an inhibitable protease (SNIP) using an FDA-approved small molecule with favorable pharmacokinetics in humans has been reported to validate no side effects and to outperform constitutive CARs in multiple solid tumor models (60). These SNIP CAR T cells serve as a predictable safety switch that can stop when lethal toxicity begins during drug administration, achieving more functional, less exhausted, and reliable levels of CAR T cells. As well as the reduced drug administration controls SNIP CAR T cells to be within the therapeutic range, eliminating tumor cells with high antigen expression, while preserving healthy tissue with lower antigen expression in an on-target off-tumor toxicity mouse model (60).

Moreover, especially in GBM patients, the CRS cytotoxic response is even more critical, as further elevated intracranial pressure in patients associated with swollen mass due to the effect of tumor can be lethal. Dexamethasone is routinely used to treat cerebral edema in patients with CNS tumors. Some clinical studies suggest that corticosteroids have an immunosuppressive effect on the desired antitumor effects of CAR T cells, but clinical evidence remains scarce (61). Dexamethasone upregulates cytotoxic T lymphocyte-associated antigen 4 (CTLA4), an immune checkpoint receptor expressed on T cells, and blocks the CD28 costimulatory pathway, which reduces T cell proliferation and differentiation (62). Conversely, low-dose dexamethasone did not reduce CAR T cell antitumor effects in orthotopic xenograft GBM models (63). By introducing a mathematical model, it was suggested that a critical threshold of CAR T cell death concerning the proliferation rate could guide the dose and timing of CAR T cell delivery in patients administered dexamethasone (64). Future clinical studies are required to determine the effects of dexamethasone on CAR T therapy.

### External therapy

Recently, several unique approaches to interrogate CAR-mediated antitumor immunity have been reported. Newly designed controllable CAR T cells, including ultrasound (FUS) control of induced heat-shock-protein promoter (Hsp) in CD19 CAR T cells (65) and photothermal modulating an IL-15 superagonist or a natural killer group 2D ligand (NKG2DL) BiTE (66), have also been reported. These switch-CAR T cells also reduced tumor burden, did not affect the surrounding tissue, improved survival, and mitigated antigen escape. As another example, the light-switchable CAR (LiCAR) T cells exhibit allowing spatial, temporal control, and T cell-mediated antitumor therapeutic effects through real-time photo-dependent activation (67).

Furthermore, engineered oncolytic viruses (OVs) exhibit tumor selectivity, targeted transgene delivery to tumors, and desirable immunogenic properties making a promising treatment approach for solid tumors. An engineered OV expressing a non-signaling truncated CD19 (CD19t) protein for selectively delivery to tumor enables targeting by CD19-CAR T cells. Infected tumor cells with an oncolytic vaccinia virus coding for CD19t (OV19t) produces *de novo* CD19 at the cell surface and subsequent virus-mediated tumor cytolysis (68). Recently, another OV study revealed that induction of the native TCR with viral or virally encoded epitopes enhances CAR T cell proliferation, activity, and efficacy in mice with intracranial GBM tumors (69).

## Conclusion

As a breakthrough treatment for GBM, CAR T therapy is crucial, through better optimization of CAR T cells is needed. Lessons learned from T cell immunotherapies have set the new stage for the application of CAR T therapy, and it is critical to identify more selective approaches, leveraging our understanding of CAR T cell resistance and toxicity. As genetic engineering rapidly advances, we have an exciting and unique opportunity to improve outcomes for GBM patients. Further basic scientific studies of CAR T cell development involving comparisons with CAR T cells for hematological malignancies are necessary to promote the translation of immunotherapy and establish it as efficient anticancer treatment.

## Author contributions

The conception and design of the paper were performed by SIC and JY. The initial draft was completed by SIC. Editing and further drafts were performed by JY. All authors reviewed the final version of the manuscript.

## Funding

This work was supported by a grant from the National Natural Science Foundation of China (82173228).

## Acknowledgments

Figures were created using BioRender.

## Conflict of interest

The authors declare that the research was conducted in the absence of any commercial or financial relationships that could be construed as a potential conflict of interest.

## Publisher’s note

All claims expressed in this article are solely those of the authors and do not necessarily represent those of their affiliated organizations, or those of the publisher, the editors and the reviewers. Any product that may be evaluated in this article, or claim that may be made by its manufacturer, is not guaranteed or endorsed by the publisher.
